# *Securidaca inappendiculata* Polyphenol Rich Extract Counteracts Cognitive Deficits, Neuropathy, Neuroinflammation and Oxidative Stress in Diabetic Encephalopathic Rats via p38 MAPK/Nrf2/HO-1 Pathways

**DOI:** 10.3389/fphar.2021.737764

**Published:** 2021-10-18

**Authors:** Xiaojun Pang, Emmanuel Ayobami Makinde, Fredrick Nwude Eze, Opeyemi Joshua Olatunji

**Affiliations:** ^1^ Department of Neurosurgery, Affiliated Hangzhou Chest Hospital, Zhejiang University School of Medicine, Hangzhou, China; ^2^ Faculty of Thai Traditional Medicine, Prince of Songkla University, Hat Yai, Thailand; ^3^ Griffith Institute for Drug Discovery, Griffith University, Brisbane, QLD, Australia; ^4^ Faculty of Pharmaceutical Sciences, Prince of Songkla University, Hat Yai, Songkhla, Thailand

**Keywords:** diabetic encephalopathy, *Securidaca inappendiculata*, oxidative stress, inflammation, MAPK pathway, Nrf2 pathway

## Abstract

Diabetic encephalopathy is one of the serious emerging complication of diabetes. *Securidaca inappendiculata* is an important medicinal plant with excellent antioxidant and anti-inflammatory properties. This study investigated the neuroprotective effects of *S. inappendiculata* polyphenol rich extract (SiPE) against diabetic encephalopathy in rats and elucidated the potential mechanisms of action. Type 2 diabetes mellitus (T2DM) was induced using high fructose solution/intraperitoneal injection of streptozotocin and the diabetic rats were treated with SiPE (50, 100 and 200 mg/kg) for 8 weeks. Learning and memory functions were assessed using the Morris water and Y maze tests, depressive behaviour was evaluated using forced swimming and open field tests, while neuropathic pain assessment was assessed using hot plate, tail immersion and formalin tests. After the experiments, acetylcholinesterase (AChE), oxidative stress biomarkers and proinflammatory cytokines, caspase-3 and nuclear factor kappa-light-chain-enhancer of activated B (NF-κB) were determined by ELISA kits. In addition, the expression levels of p38, phospho-p38 (p-p38), nuclear factor erythroid 2–related factor 2 (Nrf2) and heme oxygenase-1 (HO-1) were determined by western blot analyses. The results indicated that SiPE administration significantly lowered blood glucose level, attenuated body weight loss, thermal/chemical hyperalgesia, improved behavioural deficit in the Morris water maze, Y maze test and reduced depressive-like behaviours. Furthermore, SiPE reduced AChE, caspase-3, NF-κB, malonaldehyde malondialdehyde levels and simultaneously increased antioxidant enzymes activity in the brain tissues of diabetic rats. SiPE administration also significantly suppressed p38 MAPK pathway and upregulated the Nrf2 pathway. The findings suggested that SiPE exerted antidiabetic encephalopathy effects via modulation of oxidative stress and inflammation.

## Introduction

Type 2 diabetes mellitus (T2DM) is a lifelong complex metabolic disease that affects several millions of individuals globally. T2DM is characterized by unrelenting rise in blood glucose levels (hyperglycemia) resulting from several factors including insulin resistance, unhealthy lifestyle and obesity (Baglietto-Vargas et al., 2016; Zeliha and Canan, 2018; Gao et al., 2021). The yearly increase in the number of T2DM patients as well as the mortality accrued to the disease is alarming, thus constituting a major global public health issue. T2DM has been linked to multiple devastating complications such as neuropathy, cardiovascular disease, nephropathy, retinopathy and encephalopathy (Olatunji et al., 2018). Diabetic encephalopathy (DE) is a microvascular complication that affects the central nervous system (CNS), specifically the brain. The clinical features associated with DE includes progressive cognitive dysfunction, electrophysiological, structural and neurochemical abnormalities, leading to degeneration of the CNS and dementia (Cai et al., 2011; Soares et al., 2012; Biessels and Despa, 2018; Chen et al., 2018). It has been demonstrated that prolonged chronic hyperglycemia and hyperlipidemia contributes to the pathogenesis of DE (Chen et al., 2021).

The etiology of DE is quite complex and it is yet to be fully comprehended, however several biochemical pathways and factors have been proposed as a potential link between hyperglycemia and DE, including oxidative stress, inflammation, glucotoxicity, metal accumulation and lipotoxicity. Oxidative stress is thought to be the key mechanism in the initiation and progression of DE (Soares et al., 2012; Biessels and Despa, 2018; Erukainure et al., 2019). The brain is particularly vulnerable to reactive oxygen species (ROS) and oxidative insult due to its high demand for oxygen and glucose for energy generation through oxidative metabolic processes (McCall, 2004). As such, the increase in blood glucose level experienced during diabetes increases oxidative metabolism in the brain leading to uncontrolled generation of ROS and other reactive radicals, resulting in oxidative stress and eventually brain damage (Rossetti et al., 1990). In addition, the brain is abundant in redox active metals and polyunsaturated fatty acids, which actively participate in catalysing ROS formation and substrates for lipid peroxidation, respectively. Furthermore, the low levels of endogenous antioxidant machineries in the brain makes it almost impossible to effectively eliminate the excessive ROS and oxidative stress formed due to hyperglycemia (Smit et al., 1991; Su et al., 2004). With the increase in the incidence of diabetes, developing potent agents with strong antioxidant and anti-inflammatory properties could be a breakthrough in ameliorating diabetes and DE.

Natural products, especially medicinal plants have been extensively explored due to their potential applicability and prospects in the management of diabetes and diabetic complications. The recent surge in the use of medicinal plant as antidiabetic agents has been attributed to their low toxicity as well as their perceived efficacy (Olatunji et al., 2018; Makinde et al., 2020). *Securidaca inappendiculata* Hassk (SI) is a plant native to tropical America and it is widely grown in the tropical regions of China, especially Guangdong, Guangxi, Hainan and Yunnan provinces. SI is extensively used in Chinese folk medicine for treating inflammatory related diseases particularly rheumatic arthritis (Wang et al., 2021). In addition to its anti-inflammatory effects, SI has been reported to possess other pharmacological effects including antidiabetic, antioxidant, anticancer, hepatoprotective and cytotoxic effects (Zha et al., 2015; Ji et al., 2019; Olatunji et al., 2021). The phytochemical constituents in SI have been reported to include xanthones, neolignan glycosides and triterpene saponins (Zuo et al., 2014a; Ji et al., 2019). Our previous studies indicated that SI displayed robust antidiabetic and antioxidant effects in T2DM rats (Olatunji et al., 2021), however, there are no previous studies on the ameliorative role of SI on diabetic complications, especially DE. As such, this present study investigated the neuroprotective effects of SI in high fructose and streptozotocin induced diabetic rats. Concomitantly, given the role of oxidative damage and inflammation in T2DM and DE, we evaluated the impact of the treatment on oxidative and inflammatory markers in the brain tissues of the diabetic rats.

## Materials and Methods

### Chemicals and Reagents

Streptozotocin (STZ) was obtained from Alfa Aesar (Massachusetts, United States). ELISA kits for the estimation of proinflammatory cytokines (TNF-α, IL-6 and IL-1β) were procured from Abcam (Cambridge, United Kingdom), while biochemical kits for the estimation of antioxidants enzymes and malondialdehyde levels were purchased from Abbkine Scientific (Wuhan, China). All other chemicals used were of analytical grade.

### Preparation of Plant Extract

The dried stem samples of *Securidaca inappendiculata* Hassk was procured from Chinese Medicinal Herbs Market, Bozhou, Anhui Province, China. The plant samples was authenticated by Associate Professor Dr Jian Zuo, Department of Traditional Chinese Medicine, Wannan Medical College, Wuhu, China. A voucher specimen was deposited at the herbarium of the college. The extraction protocol for the plant material was according to our previous report (Olatunji et al., 2021). The dried extract (SiPE) was preserved at 4°C for further use.

### Animals

All the protocols used in the animal experiment were thoroughly reviewed and approved by the animal ethics committee of Prince of Songkla University (ethics approval number: 2562-04-051). Male 6 weeks old Wistar rats (160 ± 20 g) used in this study were supplied by Siam Nobura International Animal Company (Bangkok, Thailand). The rats were housed in specific pathogen-free animal house laboratory with six rats per cage and acclimatized for 1 week before the start of the experiment. During the acclimatization period, the rats were offered unlimited access to normal rat chow and water. Thereafter, the animals were divided into two groups; one group with six rats were fed with normal rat chow and water for 4 weeks (normal control animals; NCA), while the other group comprising of 24 rats were also fed with normal rat chow and 30% fructose solution for 4 weeks. Experimental diabetes was induced in 12 h fasted rats by intraperitoneal injection of STZ (35 mg/kg) dissolved in citrate buffer (pH 4.5). Diabetes was confirmed after 72 h of STZ injection by applying a drop of blood from the tail tip of the rats on glucose test strips (Accu-Check Guide, Roche). Rats with fasting blood glucose (FBG) level above 13.9 mmol/L were regarded as successful modelled diabetic rats and were further assigned into four groups:

1) DECA group (diabetic animals treated with normal saline).
2) SiPE50 group (diabetic animals treated with SiPE at 50 mg/kg).
3) SiPE100 group (diabetic animals treated with SiPE at 100 mg/kg).
4) SiPE200 group (diabetic animals treated with SiPE at 200 mg/kg).

**FIGURE 1:**
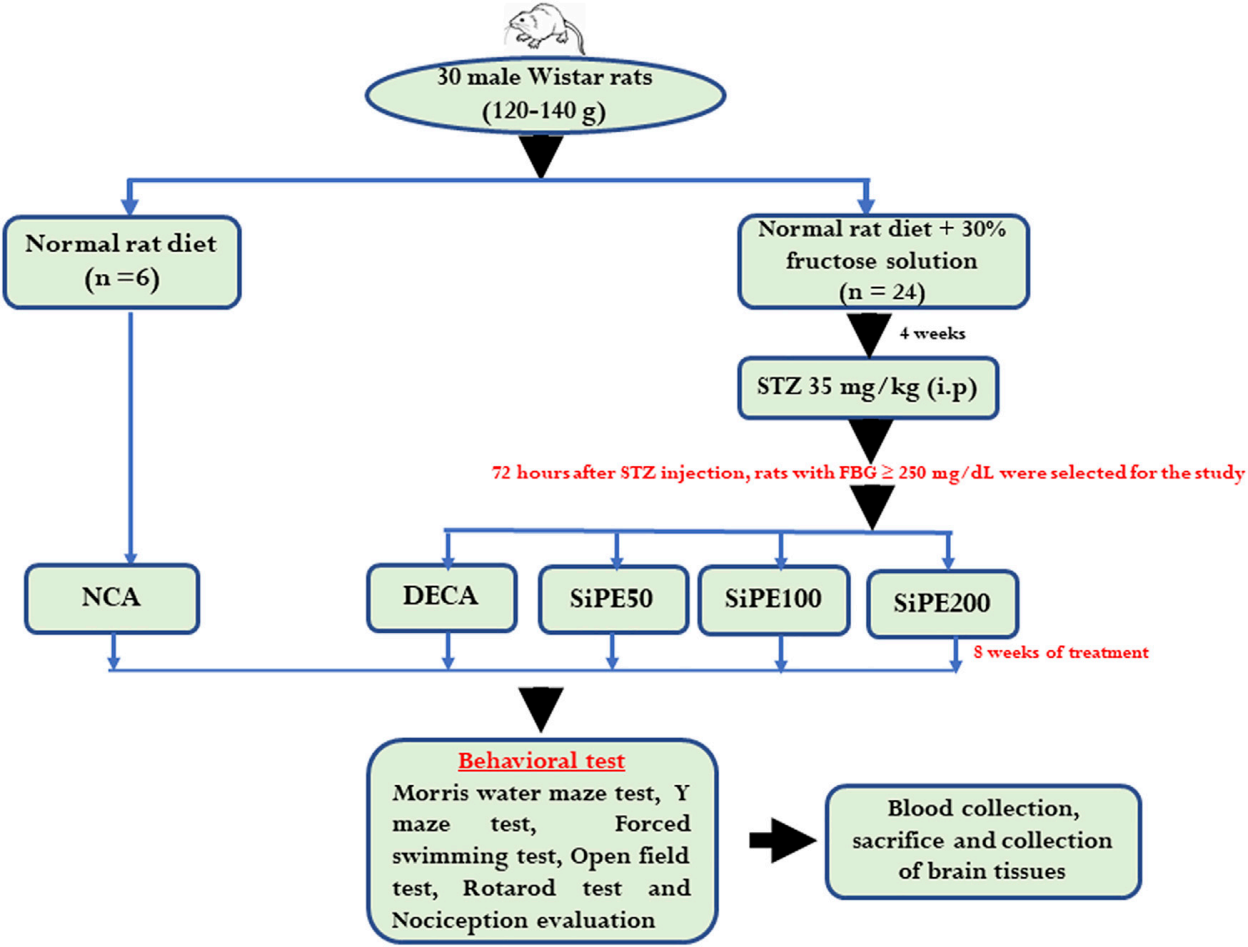
Schematic diagram showing the experimental protocol.

Oral treatment with SiPE was initiated immediately after confirmation of diabetes for 56 days (8 weeks). The choice of the doses of SiPE used was based on previous reports (Zuo et al., 2014c; Olatunji et al., 2021). The fasting blood glucose level was measured once a week with an Accu Check Guide glucometer, while changes in the body weight gain of all the animals was also measured once a week with a weighting balance. After the treatment schedule, the rats were subjected to series of behavioural test to evaluate cognitive impairment, neuropathy and motor coordination (Figure 1).

### Behavioural Studies

#### Morris Water Maze Test

The procedures and dimension of the circular pool used in the experiment was based on previously reported method of Chen et al. (2021). The temperature of the water was maintained at 25 ± 2°C throughout the experiment. The test was performed four times a day, with 30 min intervals between each test for five consecutive days. The animals were subjected to 4 days of acquisition trial, during this period the height of the escape platform was arranged to be 1 cm below the water surface. For each session during the escape latency test (days 1-4), the animals were randomly placed into the pool wall from different positions (four quadrants A, B, C and D) in succession with their heads raised up and allowed to adapt to the surface of the water for 1-2 s before been gently released to swim freely to locate the submerged platform. The time taken for the rats to locate the position of the submerged escape platform was recorded within 60 s. The rats that were able to locate the escape platform within the allotted time were allowed to stay on the platform for an additional 10 s before they were returned to their respective cages. Otherwise, the rats that were unable to locate the position of the platform within the allotted 60 s were gradually guided to the position of the platform and were also allowed to remain on the platform for 10 s. On the fifth day of the experiment (exploratory experiment) the hidden platform was removed and the time spent by each rats in the target quadrant within 60 s was recorded.

#### Y Maze Test

The Y maze test was used to evaluate spontaneous alternation of recognition using previously described method (Olasehinde et al., 2020). The maze was made up of three similar arms (1, 2, 3) with dimensions of 35 cm long, 30 cm high and 15 cm wide, stationed at equal angles. The rats were introduced into the maze from the end of one arm and allowed to freely navigate through the maze for 5 min. Spontaneous alternation was evaluated by the pattern of complete entry into each arm (the rat’s hind paws goes entirely into the arm). The frequency of alternation into the arms was recorded based on successive entries into the three arms on overlapping triplet sets (123, 231, 312 … ).

#### Forced Swimming Test

The forced swimming test was accessed based on previously reported method (Mu et al., 2020; Tsafack et al., 2021). The swimming apparatus consisted of a transparent cylinder shaped container (30 cm × 45 cm) filled with water at 25°C to a depth of 25 cm. In the pre-test training session, the rats were individually placed in the cylinder and forced to swim for 15 min. After 24 h, the animals were subjected to a 5 min session of the forced swimming. The total immobility and swimming time was recorded. Immobility time was defined as the period the rats stopped swimming, struggling and remained afloat motionlessly or only the movements necessary to keep their head above the water surface.

#### Open Field Test

The open field test (OFT) was performed using previously described method of Santiago et al. (2010). The rats were placed in the middle of an open rectangular field with floors divided into six rectangular units. The number of crossings with all four paws and rearing with the front paws was recorded for 5 min.

#### Rotarod Performance Test

The rotarod test was used for evaluating balance and motor coordination according to previous study (Shoaib et al., 2019; Saraswat et al., 2020). The rotarod apparatus consisted of a panel of rod rotating at a maximum speed of 15 rpm. The animals were allowed three sessions of training for three consecutive days. During the training session, the animals were placed on a rotating rod accelerating from 0–15 rpm and the ability of the rats to remain on the rotating rod was recorded. The animals that remained on the rotating rod by the end of the cut of time limit were given a maximum score of 600 s.

### Nociception Evaluation

Pain perception and pain thresholds in the treated rats was evaluated using the hot plate, tail immersion and formalin test. The tail immersion test was carried out using a water bath maintained at 52°C. The tails of the rats were submerged in a hot water bath set at 52.5 ± 0.5°C until the rats displays the first response of either tail flicking, withdrawal or struggle. A cut off time of 12 s was set. Shortening of the tail withdrawal time is indicative of hyperalgesia (Kishore et al., 2017). The hot plate test was performed on a hot plate set at 52 ± 0.5°C. The animals were placed on the hotplate apparatus and the latency of the first sign of either paw licking, shaking or jumping was perceived as an index of pain threshold. A cut-off time of 60 s was set to avoid tissue damage.

The formalin test was evaluated by injecting 50 µL of 2.5% formalin under the plantar dorsal surface of the right hind paw of each of the rats. The rats were placed inside a transparent open glass container. The scoring of the nociceptive behaviours started immediately after formalin injection and continued for 30 min. The early phase of the pain-related behaviours (including severe licking, biting, lifting) were recorded for the first 5 min after formalin administration, followed by a brief quiescent period and then a late phase of sustained tonic pain period (15-30 min).

### Organ Collection

After the behavioural test, the rats were fasted overnight and euthanized with an intraperitoneal injection of thiopental sodium (150 mg/kg). The brain tissues of the animals were rapidly excised, washed with normal saline to remove residual blood, weighed and a small portion of the brain was preserved in 10% buffered formalin for histopathological analysis, while the remaining part was homogenised in phosphate buffer, centrifuged at 3,500 rpm for 15 min at 4°C. The supernatant obtained was thereafter preserved at −80°C for further biochemical analysis.

### Hematoxylin and Eosin Staining

The formalin preserved brain tissues were dehydrated in graded series of alcohol, embedded in paraffin and cut into thickness of 5 μm. The section was further stained with hematoxylin and eosin and slides were visualized under light microscope.

### Determination of Acetylcholinesterase Activity in the Brain

The AchE activity in the brain tissue homogenates was determined as previously described by Ellman’s method (Ellman et al., 1961).

### Determination of Oxidative Stress Biomarkers in the Brain

The concentration of glutathione peroxidase (GPx), glutathione (GSH), superoxide dismutase (SOD), catalase (CAT) and lipid peroxidation product malonaldehyde (MDA) in the brain tissues homogenate were detected using biochemical assay kits from Abbkine Scientific Biotechnology (Wuhan, China) following the manufacturers protocol.

### Determination of Proinflammatory Biomarkers in the Brain

The levels of proinflammatory cytokines including tumor necrosis factor alpha (TNF-α), interleukin 6 (IL-6) and interleukin one beta (IL-1β) were determined using ELISA kits from Abcam (Cambridge, United Kingdom) following the procedures of the manufacturer.

### Western Blot Analysis

Protein expression were determined using western blot analysis. The brain tissues were homogenised in ice-cold RIPA buffer and the protein content was determined using a BCA protein assay kit (Beyotime Biotechnology, Shanghai, China). Equal amount of proteins were electrophoresed on 10% sodium dodecyl sulfatepolyacrylamide gel electrophoresis (SDS-PAGE) and subsequently transferred to nitrocellulose membranes. The membranes were blocked with 5% non-fat milk for 1 h. Thereafter, the membranes were incubated overnight with primary antibodies: p-p38, p38, Nrf2 and HO-1 (Abcam, Cambridge, United Kingdom). at 4°C overnight. After washing with tris-buffered saline, the membranes were further incubated with horseradish-peroxidase conjugated secondary antibodies for 2 h at room temperature. Finally enhanced chemiluminescence reagent (ECL) was used to visualize the bands and bands were analyzed densitometrically using Image J.

### Statistical Analysis

Data were analysed using GraphPad Prism 5 (GraphPad, San Diego, CA, United States) and the results were reported as mean ± SD. Statistical significance was determined using one-way ANOVA together with Newman-Keuls multiple comparison test. Statistical significance was set at *p* < 0.05.

## Results

### Effects of SiPE on FBG, Body Weight Gain and Brain Weight

**FIGURE 2:**
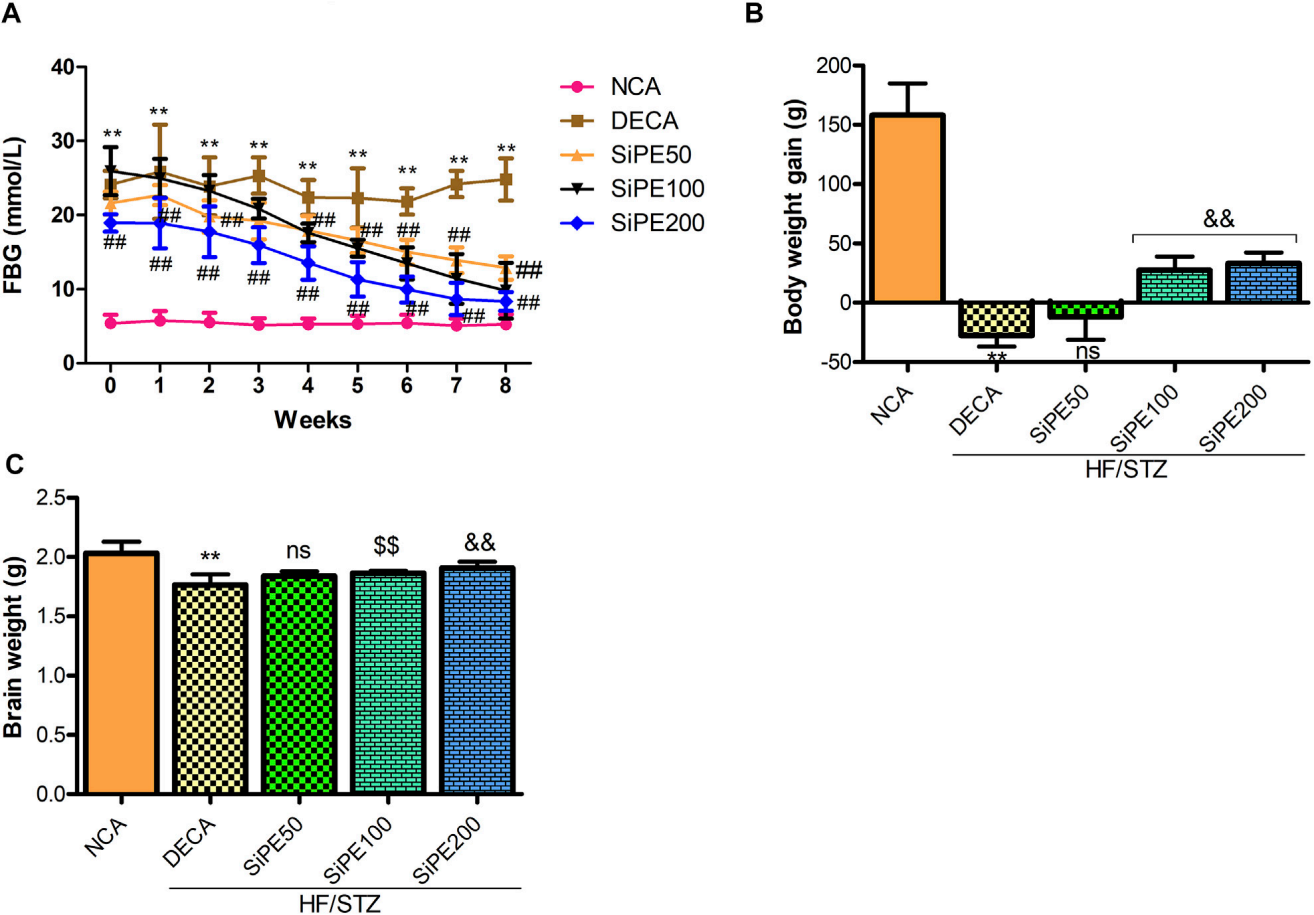
Effect of SiPE treatment on biochemical features in HF/STZ-induced diabetic encephalopathic rats. SiPE treatment at dose of 50, 100 or 200 mg/kg significantly reduced **(A)** fasting blood glucose levels. SiPE treatment at dose of 100 or 200 mg/kg significantly **(B)** improved body weight gain and **(C)** brain weight compared to DECA group. Data are presented as mean ± SD (*n* = 6) and one-way ANOVA test was applied. **p < 0.001 indicates statistical significance compared to NCA group. ##*p* < 0.001, &&*p* < 0.01 and $$*p* < 0.05 indicate statistical significance compared to DECA group. ns: not significant compared to DECA group.

As shown in Figure 2A, the rats administered with STZ had FBG levels >13.9 mmol/L which were significantly higher (*p* < 0.01) than the corresponding FBG level of rats in the NCA group. Whereas, significant and dose dependent decrease was observed in the final FBG levels of rats that received SiPE (50, 100 and 200 mg/kg) after 8 weeks of supplementation when compared to the untreated DECA group (Figure 2A). Furthermore, as indicated in Figure 2B, the rats in the DECA group showed significant weight loss compared with the NCA group (*p* < 0.01). After 8 weeks of treatment with SiPE, there were significantly improvement in the weight of rats compared with the DECA group. Furthermore, the diabetic rats treated with SiPE showed increased brain weight when compared to DECA control group (Figure 2C).

### SiPE Improved Learning and Memory Abilities of Diabetes Mellitus Rats

**FIGURE 3:**
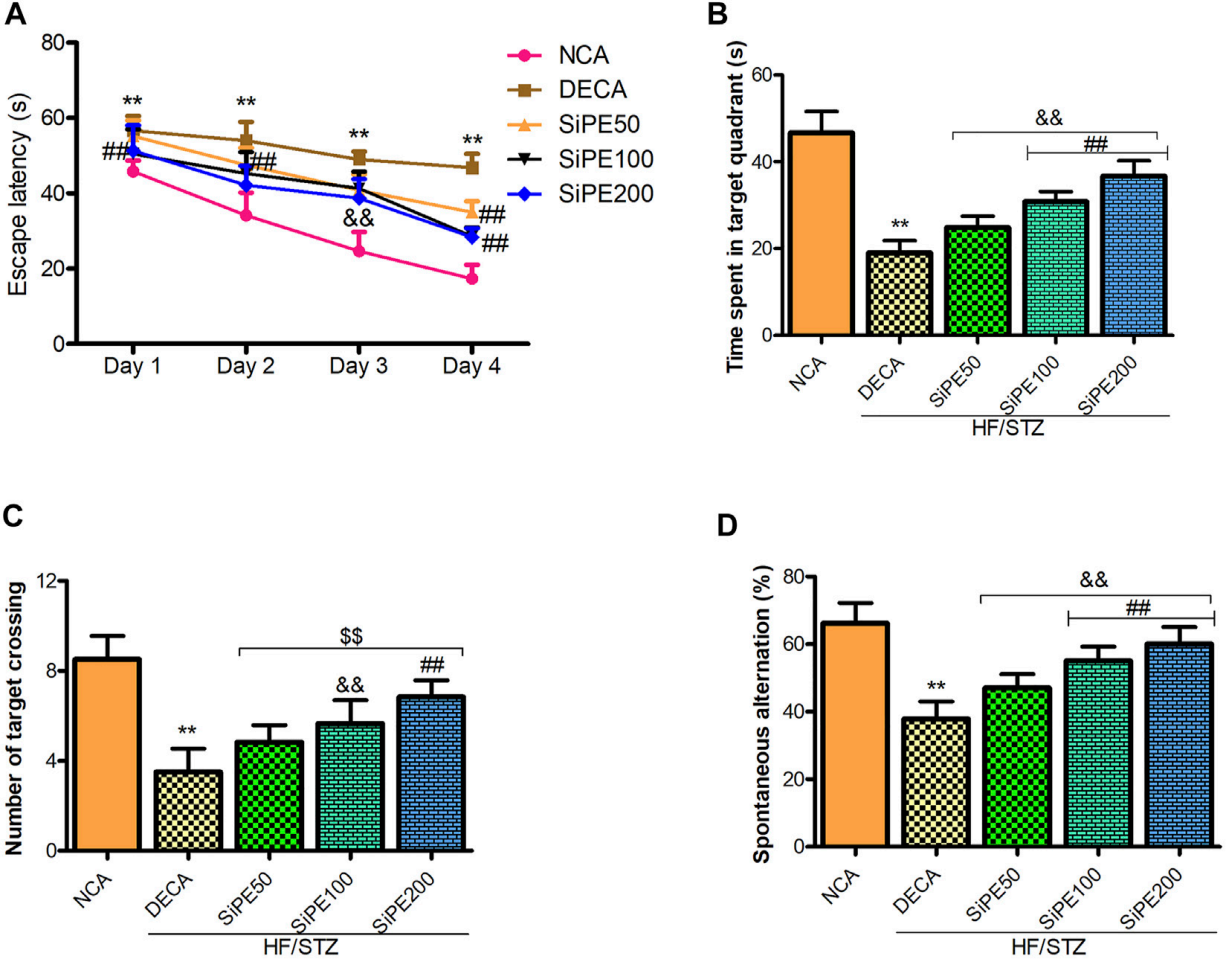
Effect of SiPE treatment on behavioural parameters in HF/STZ-induced diabetic encephalopathic rats. SiPE treatment at dose of 50, 100 or 200 mg/kg significantly improved **(A)** escape latency, **(B)** time spent in the target quadrant, **(C)** number of target crossing and **(D)** spontaneous alternation compared to DECA group. Data are presented as mean ± SD (*n* = 6) and one-way ANOVA test was applied. **p < 0.001 indicates statistical significance compared to NCA group. ##*p* < 0.001, &&*p* < 0.01 and $$*p* < 0.05 indicate statistical significance compared to DECA group.

Next, we performed Morris water maze and Y maze test to evaluate the effect of SiPE on memory and learning impairments in T2DM rats. As indicated in Figure 3A, the rats in the DECA group spent significantly longer time to locate the hidden platform during the 4 days trial period compared to the NCA group (Figure 3A). Whereas, the escape latency time of the SiPE treated groups were significantly shorter compared to the DECA group (Figure 3A). In the space exploration test, it was observed that the DECA rats spent significantly shorter time in the target quadrant when compared to the NCA rats. Supplementation with SiPE significantly and dose dependently increased the time spent in the target quadrant when compared to the DECA group (Figure 3B). Additionally, the number of target crossing of rats in the DECA group was significantly reduced compared to the NCA group, whereas, the diabetic rats treated with SiPE showed increased number of target quadrant crossing when compared to the DECA group (Figure 3C).

In the Y maze test, there was a significant decrease in the percentage of spontaneous alternations in the DECA group compared to the NCA group (Figure 3D). On the contrary, the reduction in percentage of spontaneous alternations observed in the DECA group was obviously reversed in the SiPE treated groups (50, 100 and 200 mg/kg) (*p* < 0.01; Figure 3D).

### Effect of SiPE on Immobility Time in Forced Swimming Test and Open Field Test

**FIGURE 4:**
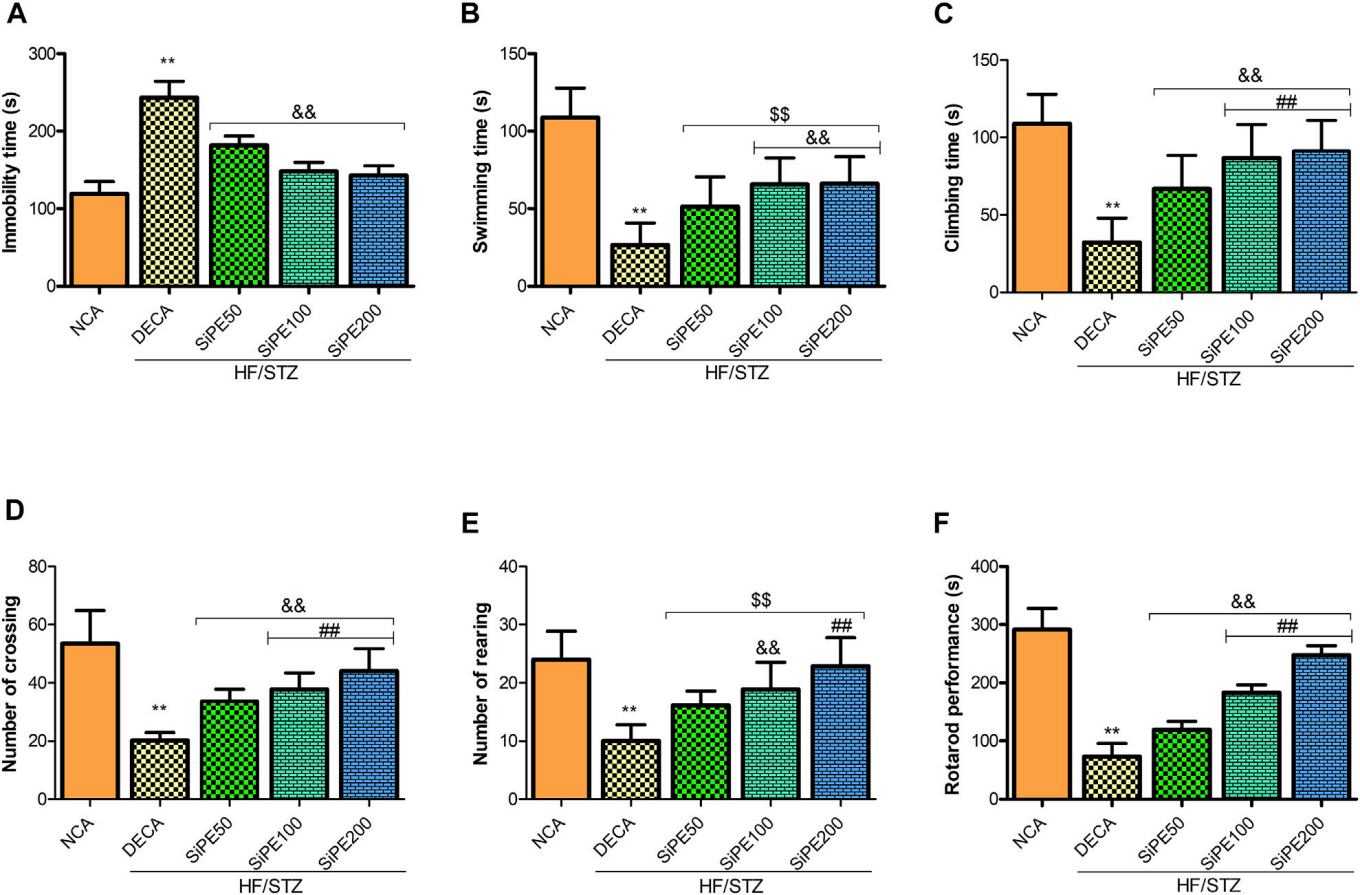
Effect of SiPE treatment on depressive-like behaviours and motor coordination in HF/STZ-induced diabetic encephalopathic rats. SiPE treatment at dose of 50, 100 or 200 mg/kg significantly ameliorated **(A)** immobility time, **(B)** swimming time, **(C)** climbing time, **(D)** number of crossing, **(E)** number of rearing and **(F)** rotarod performance compared to DECA group. Data are presented as mean ± SD (*n* = 5–6) and one-way ANOVA test was applied. **p < 0.001 indicates statistical significance compared to NCA group. ##*p* < 0.001, &&*p* < 0.01 and $$*p* < 0.05 indicate statistical significance compared to DECA group.

Figures 4A–C shows the effect of SiPE on immobility time, swimming and climbing time in the FST. The DECA rats showed significantly increased immobile time, with a corresponding decrease in the time spent swimming and climbing when compared to the NCA rats. In contrast, the diabetic rats that received SiPE showed significantly reduced immobility time as well as increased swimming and climbing time in comparison to the rats in the DECA group (Figures 4A–C). Additionally, the number of crossing and rearing of the DECA group was significantly decreased when compared to the NCA and SiPE treated rats (Figures 4D,E).

### Effect of SiPE on Motor Coordination

As shown in Figure 4F, the DECA rats showed significant reduction in endurance and motor coordination as indicated by the reduced time spent on the rotating rod when compared to the time spent by the NCA rats. In contrast, treatment with SiPE significantly and dose dependently increased the time spent on the rotarod compared to the DECA rats (Figure 4F).

### Effects of SiPE on Pain Scores

**FIGURE 5:**
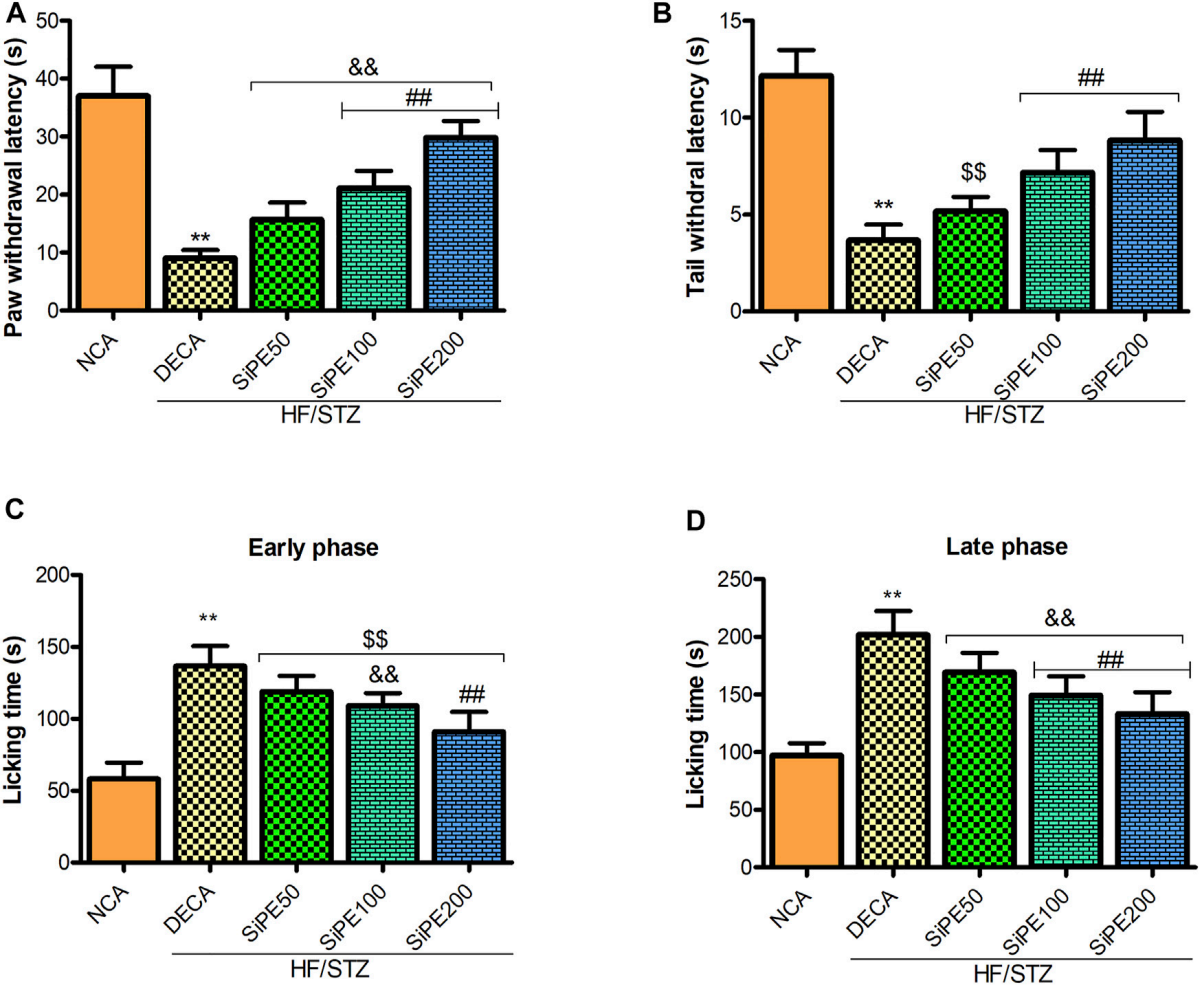
Effect of SiPE treatment on thermal and chemical hyperalgesia in HF/STZ-induced diabetic encephalopathic rats. SiPE treatment at dose of 50, 100 or 200 mg/kg significantly ameliorated **(A)** paw withdrawal latency, **(B)** tail withdrawal latency, **(C)** paw licking time (early phase) and **(D)** paw licking time (late phase) compared to DECA group. Data are presented as mean ± SD (*n* = 6) and one-way ANOVA test was applied. **p < 0.001 indicates statistical significance compared to NCA group. ##*p* < 0.001, &&*p* < 0.01 and $$*p* < 0.05 indicate statistical significance compared to DECA group.

As shown in Figure 5, compared with the NCA group, the pain perception of the DECA group was significantly increased as indicated by marked reduction in the tail and paw withdrawal latencies in the tail immersion and hot plate test, respectively (Figures 5A,B). In addition, the nociceptive reaction of the untreated DECA group in the formalin test was significantly increased as indicated by high incidence of pain response (paw licking, biting and biting) in both the early and late phases (Figures 5C,D). Whereas, administration of SiPE significantly reduced both thermal and chemical induced hyperalgesia as portrayed by the increased level of pain perception in the treated rats (Figures 5A–D).

### Effects of SiPE on Oxidative Stress Biomarkers in the Brain of Diabetes Mellitus Rats

**FIGURE 6:**
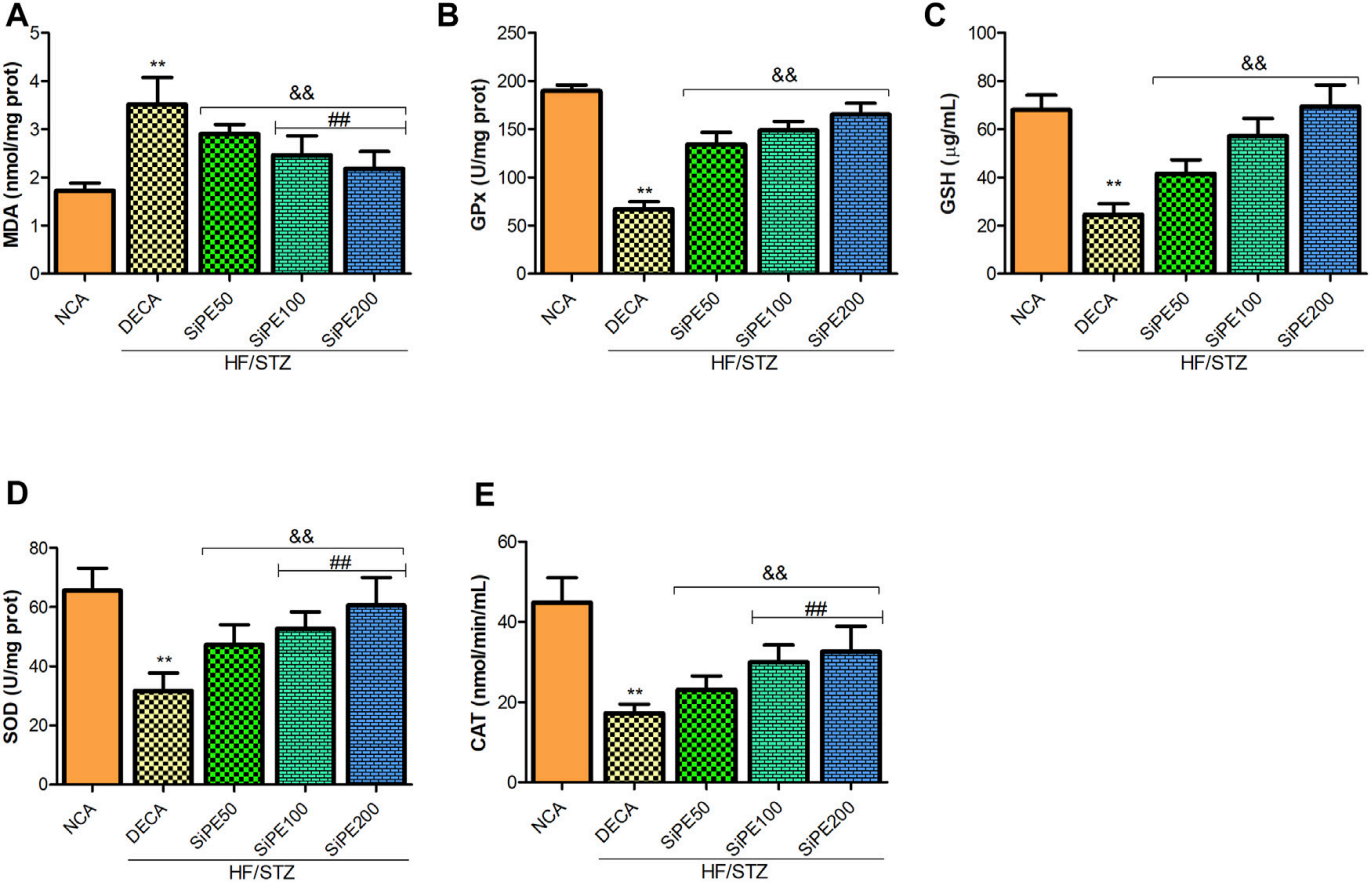
Effect of SiPE treatment on oxidative stress/antioxidant biomarkers in HF/STZ-induced diabetic encephalopathic rats. SiPE treatment at doses of 50, 100 or 200 mg/kg caused significant decrease in **(A)** brain malondialdehyde (MDA) levels compared to DECA group. SiPE treatment reversed HF/STZ induced reduction in **(B)** brain glutathione peroxidase (GPx), **(C)** reduced glutathione (GSH), **(D)** superoxide dismutase (SOD) and **(E)** catalase (CAT). Data are presented as mean ± SD (*n* = 6) and one-way ANOVA test was applied. **p < 0.001 indicates statistical significance compared to NCA group. ##*p* < 0.001, &&*p* < 0.01 and $$*p* < 0.05 indicate statistical significance compared to DECA group.

As shown in Figure 6A, the level of lipid peroxidation (MDA) in the brain tissues of the DECA group was significantly higher than the NCA group, whereas MDA level was significantly lowered in SiPE treated groups compared with the DECA group. The activities of GPx, GSH, CAT and SOD in the brain of DECA rats were also observed to be notably reduced in comparison to the NCA rats (Figures 6B–E). On the contrary, the activities of the aforementioned antioxidant enzymes in the SiPE treated groups were significantly upregulated when juxtaposed with the DECA group (Figures 6A–E).

### Effects of SiPE on Proinflammatory Cytokines in the Brain of Diabetes Mellitus Rats

**FIGURE 7:**
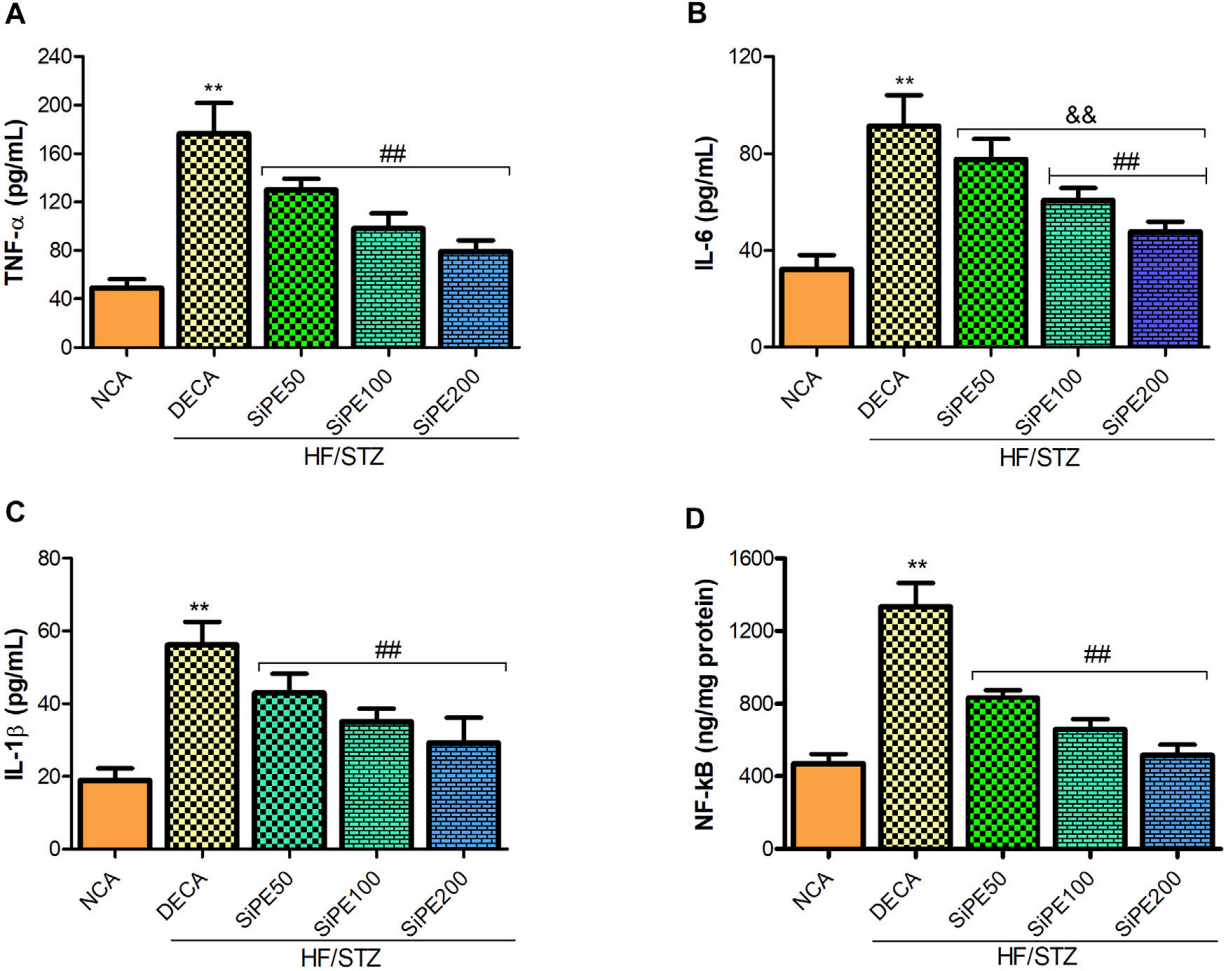
Effect of SiPE treatment on inflammatory mediators in HF/STZ-induced diabetic encephalopathic rats. SiPE treatment at doses of 50, 100 or 200 mg/kg caused significant decrease in **(A)** brain tumor necrosis factor alpha (TNF-α), **(B)** interleukin 6 (IL-6), **(C)** interleukin one beta (IL-1β) and **(D)** nuclear factor kappa-light-chain-enhancer of activated B (NF-κB) compared to DECA group. Data are presented as mean ± SD (*n* = 5–6) and one-way ANOVA test was applied. **p < 0.001 indicates statistical significance compared to NCA group. ##*p* < 0.001, &&*p* < 0.01 and $$*p* < 0.05 indicate statistical significance compared to DECA group.

We investigated the effect of SiPE on proinflammatory cytokines in the brain tissues of the treated rats. Figures 7A–C indicated that the DECA group showed increased concentration of TNF-α, IL-6 and IL-1β compared to the NCA group, while the increased levels of proinflammatory cytokines were significantly reduced by SiPE treatment (Figures 7A–C). Additionally, NF-κB levels was significantly increased in the brain tissues of the DECA rats compared with the NCA group, while the levels was dose dependently and significantly reduced after treatment with SiPE (Figure 7D).

### Effects of SiPE on AChE and Caspase-3

**FIGURE 8:**
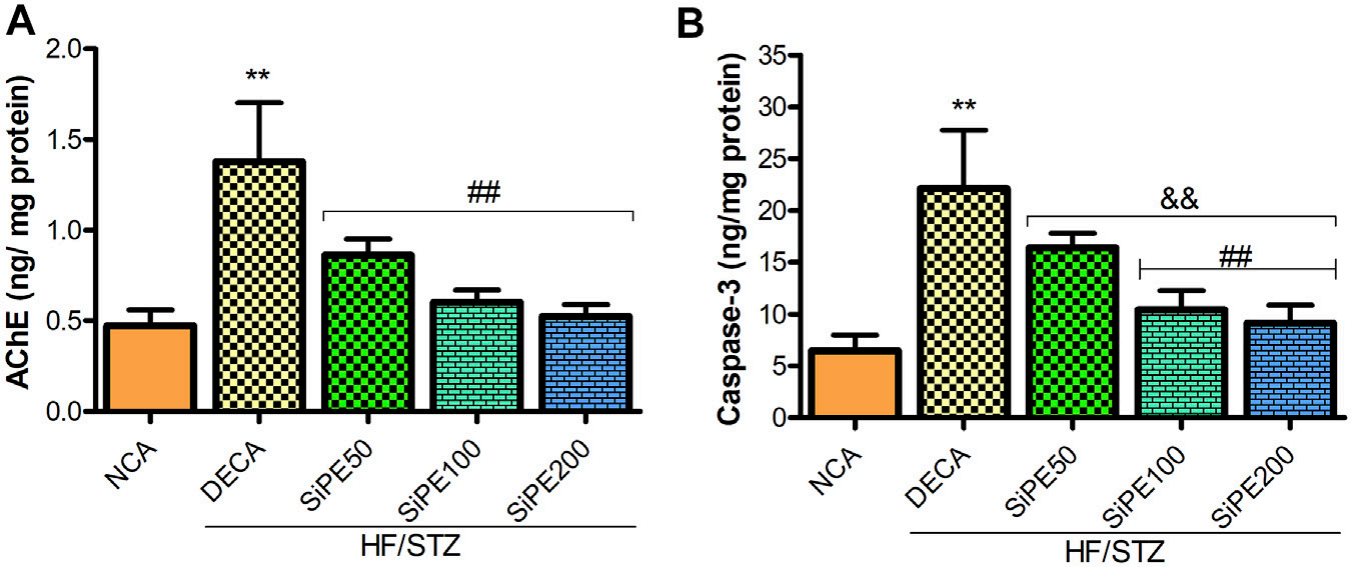
Effect of SiPE treatment on biochemical parameters in HF/STZ-induced diabetic encephalopathic rats. SiPE treatment at doses of 50, 100 or 200 mg/kg caused significant reduction in **(A)** acetylcholinesterase (AChE) and **(B)** caspase-3 levels compared to DECA group. Data are presented as mean ± SD (*n* = 6) and one-way ANOVA test was applied. **p < 0.001 indicates statistical significance compared to NCA group. ##*p* < 0.001, && *p* < 0.01 and $$*p* < 0.05 indicate statistical significance compared to DECA group.

In the current study, the levels of AChE and caspase-3 in the brain of the untreated DECA group was significantly heightened by approximately 3 and 4 folds respectively when compared with the NCA group. Nevertheless, the administration of SiPE attenuated AChE and caspase-3 levels compared to the DECA group (Figures 8A,B).

### Effects of SiPE on Histopathological Analysis Using Hematoxylin and Eeosin Staining

**FIGURE 9:**
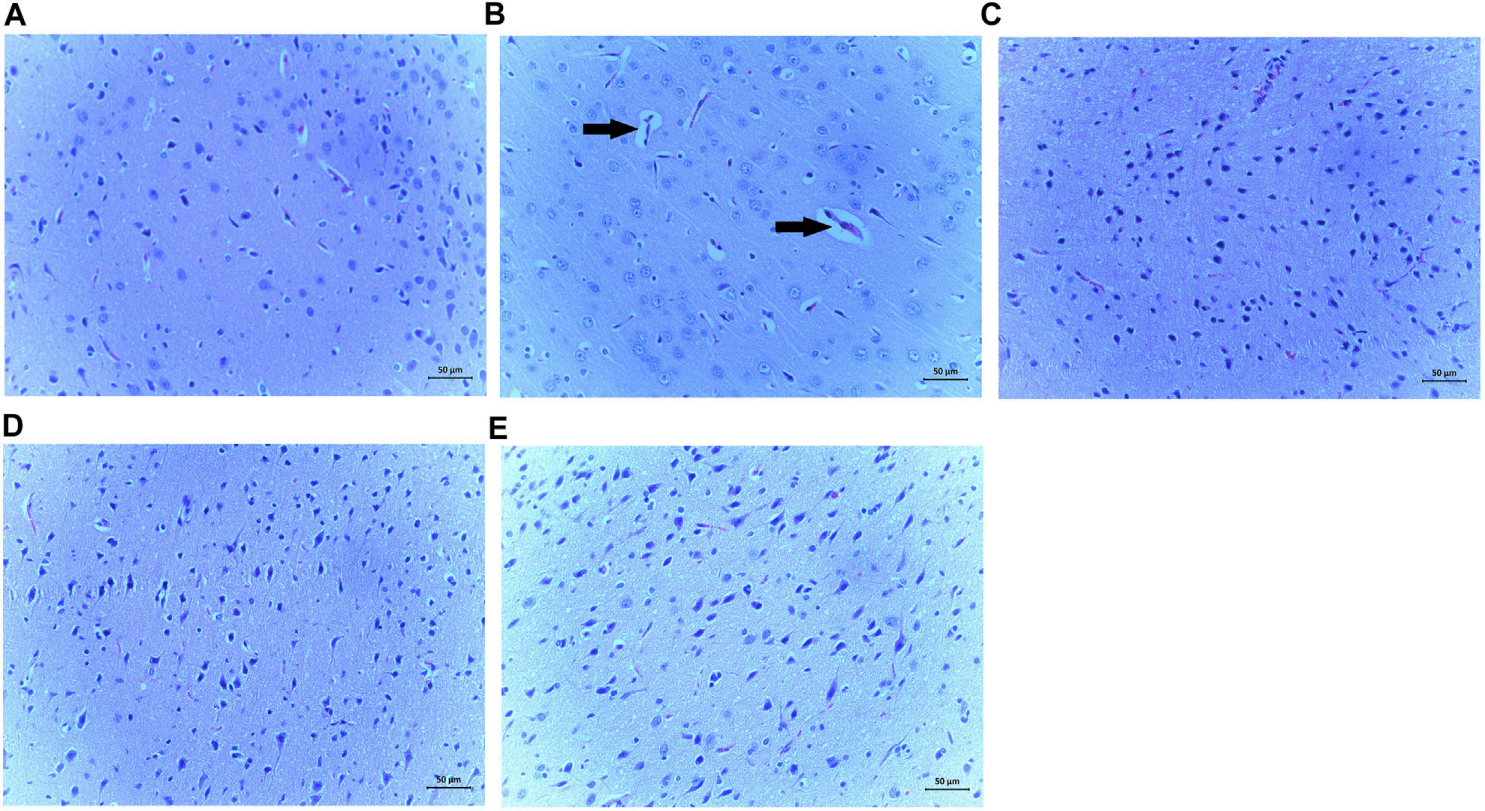
Effect of SiPE treatment on histopathological features in HF/STZ-induced diabetic encephalopathic rats. Representative images of H&E staining of brain tissues showing **(A)** NCA group, **(B)** DECA group, **(C)** SiPE50 group, **(D)** SiPE100 group and **(E)** SiPE200 group. (magnification ×200, scale bar = 50 μm).

The results from the representative H&E staining of the brain tissues of the treated is shown in Figure 9. The DECA control representative image displayed obvious pathological alterations including glial proliferation and neuron apoptosis (Figure 9B). However, changes in brain architecture were abrogated in the rats treated with SiPE compared to the DECA rats (Figure 89C-E).

### Effects of SiPE on MAPK-p38 Signaling Pathway

**FIGURE 10:**
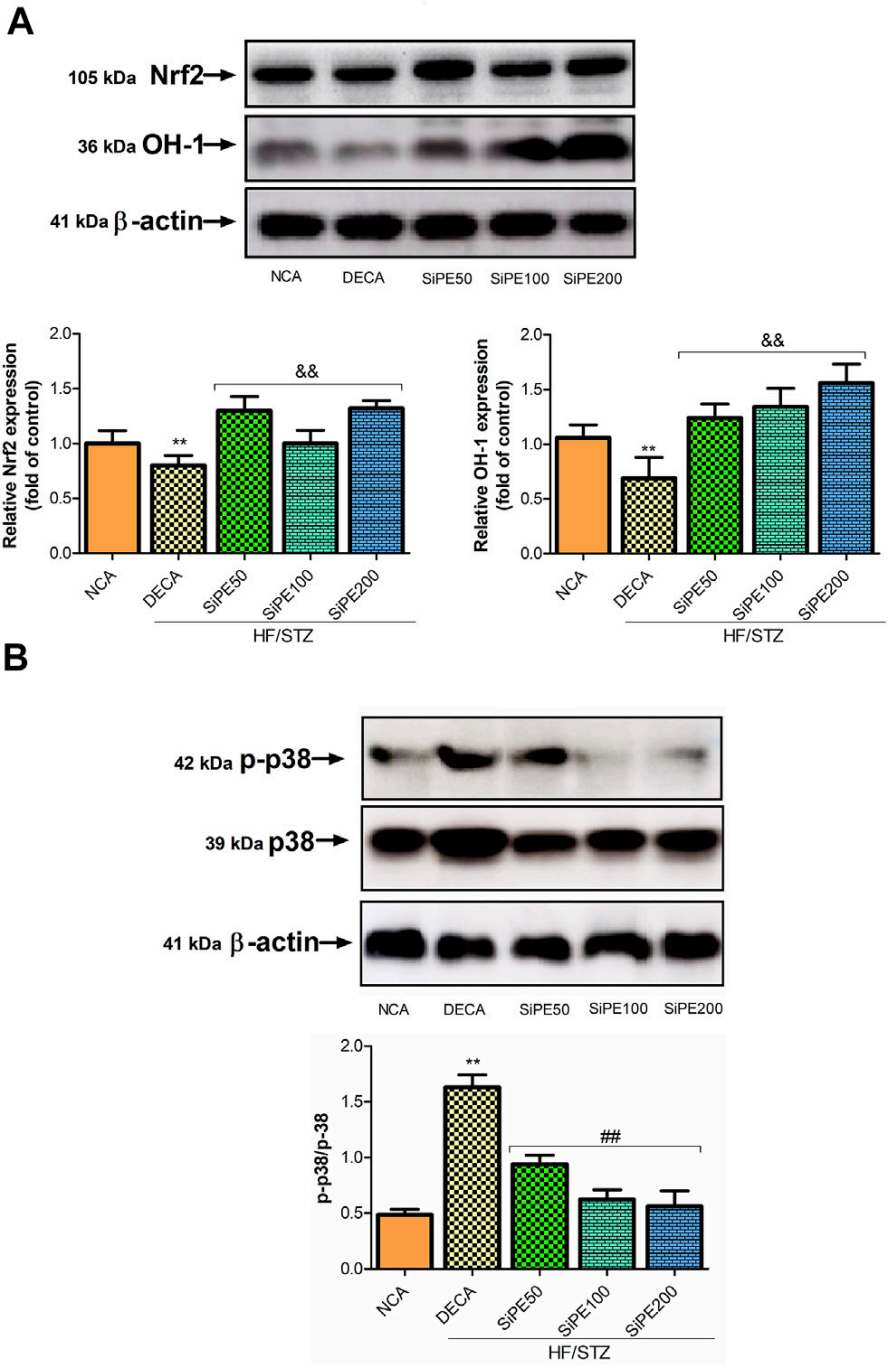
Protein expressions of p-38, p-p38, Nrf2 and HO-1 in the brain tissues from NCA, DECA and SiPE treated groups. Data are presented as mean ± SD (*n* = 6) and one-way ANOVA test was applied. **p < 0.001 indicates statistical significance compared to NCA group. ##*p* < 0.001, &&*p* < 0.01 and $$*p* < 0.05 indicate statistical significance compared to DECA group.

The effects of SiPE on the expression of MAPK signaling (p38 and p-p38) are shown in Figure 10. The results indicated that p-p38 expression was significantly increased in the DECA groups, while treatment with SiPE suppressed the phosphorylation of p38 in the treated rats (Figure 10). These results suggested that SiPE suppressed the activation of p38 MAPK pathway in the brain tissues of diabetic rats.

### Effect of SiPE on Nrf2 Signaling Pathway

The effects of SiPE on Nrf2 signaling pathway in the treated rats are shown in Figure 10. The DECA group showed reduced expression of nuclear Nrf2 and HO-1 compared to the normal control group. While SiPE treated groups exhibited increased expression of Nrf2 and HO-1 compared to the DECA group.

## Discussion

DE is a complex complication arising from uncontrolled hyperglycemia and the complex nature of DE has encouraged and increased the use of alternative avenues for treating the disease. Increasing number of studies have shown that medicinal plants have shown effectiveness in regulating biochemical, neurological and behavioural alterations in diabetic models due to their multi-constituents and multitarget nature (Kim et al., 2016). This study investigated the neuroprotective effects of SiPE in diabetic rats. The results indicated that after 8 weeks of treatment, SiPE significantly improved hyperglycemia induced memory and cognitive dysfunction, hyperalgesia as well and attenuated inflammatory and oxidative stress responses in the brain tissues of the treated diabetic rats.

Increasing evidences have suggested that diabetic patients are more prone to developing Alzheimer’s disease, vascular dementia, cognitive deficits and brain atrophy (Jayaraman and Pike, 2014; Ye et al., 2018). Hyperglycemia plays a crucial role in neuronal damage through the instigation of oxidative stress sensitive pathways leading to neuronal apoptosis and damage. In addition, diabetic alterations has been largely reported to influence learning and memory function (Thakur et al., 2016; Chandrasekaran et al., 2020; Shin et al., 2021). As such, maintaining normal glycaemic control is paramount in the treatment of diabetes so as to prevent any diabetic associated complication including DE. The results from our study indicated that high fructose/STZ induced diabetes was associated with significantly high levels of FBG and reduced body weight gain which was consistent with results from other previous studies (Makinde et al., 2020; Gao et al., 2021).

There are accumulating clinical and experimental evidences that suggests that diabetes can lead to impaired cognitive function. In this current study, diabetes induced significant memory and learning impairments in the MWMT and Y-maze test, which is in accordance with previous reports (Gocmez et al., 2019; Chen et al., 2021). The occurrence of neuropathy is a common phenomenon in diabetic patients and it significantly affects neuroplasticity and cognitive retention (Tian et al., 2016; Gocmez et al., 2019). The findings from this study indicated that diabetes induced learning, memory and locomotive activity impairments were attenuated after treatment with SiPE. The rats treated with SiPE had significantly shorter escape latency as well as improved percentage alternation, suggesting improvement in spatial learning and memory.

Depression is one of the comorbidity associated with diabetes mellitus. In fact diabetes and depression have been shown to display a bifacial etiology, as patients suffering from depression have a higher risk of developing T2DM due to the activation of several pathways that can initiate insulin resistance (Champaneri et al., 2010; Deischinger et al., 2020). In addition, previous studies have illustrated that anxiety and depression are characteristic features associated with diabetic neuropathy (Mbiantcha et al., 2019; Tsafack et al., 2021). FST and OFT are the most commonly used behavioural studies for evaluating antidepressant activity of pharmacological agents (Dulawa et al., 2004; Cryan et al., 2005). A behavioural depressive state is indicated by increase in immobility time displayed by experimental animals in the OFT and FST tests (Shalam et al., 2007). In line with previous studies, we observed that diabetic rats exhibited depressive like behaviour as indicated by increased immobility time in the FST, as well as reduced locomotive activity in the OFT test when compared to the normoglycemic rats (de Morais et al., 2014, de Morais et al., 2016; Tsafack et al., 2021). SiPE administration significantly reduced the immobility time and increased the swimming time as well as the exploratory behaviour in the FST and OFT, respectively.

In this study, we observed that AChE activity in the brain of diabetic rats were significantly increased. Several studies have reported a striking and direct relationship between increased brain cholinesterase activity and impaired cognitive function in diabetes (Schmatz et al., 2009; Zarrinkalam et al., 2018; Agunloye and Oboh, 2021). The marked increase in the activity of brain AChE in the diabetic rats corroborates the results of the MWM and Y-maze test. Interestingly, treatment with SiPE significantly ameliorated AChE activity in the brain of diabetic rats. These results were consistent with previous studies on diabetes induced cognitive dysfunction (Mushtaq et al., 2014).

Diabetes has also been reported to significantly affect pain response (Kishore et al., 2018; Abo-Salem et al., 2020). Hyperalgesia, a condition that is exemplified by extreme sensitivity to painful stimuli is one of the most often encountered complication of diabetes, and it arises due to damages to peripheral nerves (Lee-Kubli et al., 2014; Hasanein et al., 2020). Motor and sensory nerve conduction dysfunction, numbness and chronic pain are characteristics features that have been well documented to correlate with chronic hyperglycemia (Edwards et al., 2008). Previous studies have also reported increased pain sensation in diabetic animal models (Kishore et al., 2017; Abo-Salem et al., 2020). In this study, marked decrease in nociceptive threshold in the hotplate and tail immersion test were observed in the diabetic rats, suggesting that the diabetic rats displayed increased hyperalgesia. Treatment with SiPE dose dependently and significantly ameliorated thermal hyperalgesia in the treated rats. Additionally, the increased occurrence of formalin elicited flinching responses in diabetic rats is indicative of abnormal responses to nociceptive stimuli, which has been extensively reported in previous studies (Kaur et al., 2017; Hasanein et al., 2020). In the present study the injection of formalin lead to a biphasic nocifensive flinching, licking or biting behaviour that was amplified in the diabetic animals. The administration of SiPE (50, 100 and 200 mg/kg) lowered the occurrence of chemical hyperalgesia during phases 1 (neurogenic phase) and 2 (inflammatory phase) of the formalin test.

Oxidative stress is a major player in the mechanism associated with diabetic cognitive decline and multiple studies have demonstrated that the pathophysiology and progression of DE is extensively mediated by free radical-induced oxidative stress (Jing et al., 2013; Gocmez et al., 2019; Chen et al., 2021). Excessive increase in oxidative stress coupled with impaired antioxidant defence attributed to uncontrolled hyperglycemia encourages the generation of ROS, which subsequently inactivates nitric oxide bioavailability in diabetes induced endothelial dysfunction, leading to impaired cerebral perfusion, neuronal damage, brain injury and diabetic induced neurodegeneration (Ceriello, 2003; Gocmez et al., 2019). In this study, the activities of SOD, GPx and CAT in the brain tissues of diabetic rats were significantly lowered, while lipid peroxidation (MDA) level was increased. These alterations in the brain oxidative stress and antioxidant defence capacity may be attributed to hyperglycemia induced over generation of ROS, which might have overshadowed the brain antioxidant homeostasis. These results were in consonance with other previous studies revealing loss or reduced brain antioxidant enzymes activity in DE models (Erukainure et al., 2019; Zhang et al., 2020). Treatment with SiPE reversed the decline in antioxidant enzymes activity, while concomitantly reducing lipid peroxidation (MDA) levels in the brain of the treated rats.

On the other hand, prolonged hyperglycemia instigated oxidative stress has also been shown to be a primary facilitator of inflammatory reactions, which aggravates the progression of cognitive decline and brain injury in diabetes. Proinflammatory cytokines including TNF-α, IL-6 and IL-1β are notable cytokines that rapidly responds to oxidative damages and they have been extensively implicated in central and peripheral inflammatory reactions in diabetes (Ola et al., 2014; Wang et al., 2020; Xianchu et al., 2021). Enhanced proinflammatory cytokines concentration directly affects synaptic activity and neurotransmission of neurons, resulting in cognitive dysfunction. In the current study, TNF-α, IL-6 and IL-1β levels were significantly increased in the brain tissues of diabetic rats, while treatment with SiPE exhibited potent anti-inflammatory prowess by significantly abating the level of these cytokines in the treated rats. These results agree with previous studies on the anti-inflammatory potentials of SiPE, especially its effects on cytokine levels in models of inflammation (Zuo et al., 2020).

Oxidative stress can activate a number of apoptotic and inflammatory pathways including caspases and NF-κB pathways, which further complicates DE. NF-κB is a vital transcriptional factor that regulates immunity and it is also extensively involved in the activation of several inflammatory cytokines including TNF-α, IL-6 and IL-1β. Emerging evidences has implicated NF-κB in the pathologies of multiple diseases including diabetes and diabetic complications (Syed et al., 2020; Chen et al., 2021). Consistent with the studies by Chen et al. (2021) and Liu et al. (2020), our study found that diabetes induced significant increase in NF-κB levels in the brain tissues of diabetic rats. Supplementation with SiPE attenuated NF-κB levels, indicating that SiPE displayed strong anti-inflammatory capacity. Furthermore, our study also observed increase in caspase 3 levels in the brain tissues of diabetic rats. Numerous literatures have established the pivotal pathological roles of oxidative stress and proinflammatory cytokines in apoptosis. Notably ROS generation and the resulting oxidative stress in the mitochondrial can lead to mitochondria dysfunction through the release of several apoptosis factors including cytochrome C and caspase 3 into the cytosol, which ultimately leads to apoptotic cell death (Kannan and Jain, 2000; Wang et al., 2020). Increased levels or over activation of apoptosis players including caspase-3 have been implicated in the pathophysiology of many chronic disorders including Alzheimer, Parkinson and diabetes (Kannan and Jain, 2000). Consistent with previous observation, our result indicated that SiPE supplementation decreased the levels of caspase-3 in the brain of diabetic rats (Tian et al., 2016).

Nrf2 is a transcriptional factor that is very sensitive to redox changes and it is responsible for promoting the expression of antioxidant genes in response to oxidative insult (Ma and Long, 2016; Xu et al., 2021). Under normal biological conditions, Nrf2 binds to Keap1, which restrict its ability to bind to antioxidant related element (ARE). However, in the event of oxidative stress, Nrf2 is released from Keap1, degraded and binds to ARE, thus activating Nrf2-target genes such as NQO1 and HO-1 (Ma and Long, 2016; Wen et al., 2019). In addition, HO-1 has been demonstrated to play a very important role in inflammation due to its suppressive ability on proinflammatory mediators (Ma and Long, 2016; Saha et al., 2020). In this present study, diabetes down regulated the expression of Nrf2/HO-1 which was in accordance with the results from previous study (Ma and Long, 2016; Xu et al., 2021). SiPE ameliorated these alterations by up-regulating Nrf2/HO-1 expressions in the brain tissues of the treated rats.

The mitogen-activated protein kinase (MAPK) pathway has been linked to a number of pathogenic responses including inflammation and oxidative stress. Supporting evidences have shown that MAPK signaling pathways are critically involved in the pathogenesis of several diabetic complications (Lu et al., 2020). Specifically, MAPK p38 pathway has been reported to be activated in diabetes (Ma et al., 2015; Zhao et al., 2021). The results from this study demonstrated that SiPE attenuated diabetes associated activation of p38 MAPK and increase in p-p38 phosphorylation.

Meanwhile, several evidences have demonstrated that plant polyphenols exhibited protective effects against several diseases including diabetes, inflammation, obesity, liver injury and neurodegenerative diseases (Daglia et al., 2014; Tang et al., 2014; Olatunji et al., 2017; Silva and Pogačnik, 2020). The considerable quantity of polyphenols in *S. inappendiculata* provides significant antioxidant and anti-inflammatory effects (Ji et al., 2021; Olatunji et al., 2021). Our previous study on the phytochemical analysis of SiPE extract using UHPLC-ESI-QTOF-MS affirmed the phenolic-richness of the plant, mainly glycosylated polyphenolic derivates, including natsudaidain 3-(4-O-3-hydroxy-3-methylglutaroylglucoside), pectolinarin, centaurein, mirificin, eriodictyol, acanthoside D, neocuscutoside C, oenanthoside A, methylpicraquassioside A, furocoumarinic acid glucoside, hydroxycinnamic acid glycoside, embelin, 5-O-methylembelin, irisxanthone and euxanthone. In addition, the extracts of SiPE have been reported to contain xanthones such as 1,3,7-trihydroxyl-xanthone, 1,3,7-trihydroxyl-2-methoxyl-xanthone, 1,7-dihydroxyl-3,4-dimethoxyl-xanthone, 1,7-dihydroxyl-xanthone, 2-hydroxyl-1,7-dimethoxyl-xanthone, and 7-hydroxyl-1,2-dimethoxyl-xanthone. These compounds have been reported to have varied therapeutic potentials, including robust antioxidant, antidiabetic, anti-inflammatory and analgesic activities (Zuo et al., 2014b; Zuo et al., 2014c; Olatunji et al., 2021). On the basis of our results, we propose that the polyphenol-rich component of SiPE may have been responsible for the potential antidiabetic encephalopathic activity observed in this study.

## Conclusion

Overall, this study illustrated that SiPE reduced blood glucose levels, attenuated learning and memory dysfunction and reduced neuronal damage in diabetic rats. The findings further suggested that the ameliorative impact of SiPE on diabetic encephalopathy may be attributed to its ability to reduce oxidative stress and pro-inflammatory cytokines, as well as modulate MAPK p38 signaling pathway and the promotion of Nrf2 signaling pathway.

## Data Availability

The original contributions presented in the study are included in the article/Supplementary Material, further inquiries can be directed to the corresponding author.
